# Parents’ beliefs and knowledge about the management of acute otitis media: a qualitative study

**DOI:** 10.1186/s12875-015-0297-7

**Published:** 2015-07-07

**Authors:** Malene Plejdrup Hansen, Janine Howlett, Chris Del Mar, Tammy C. Hoffmann

**Affiliations:** Centre for Research in Evidence-Based Practice, Faculty of Health Sciences and Medicine, Bond University, Gold Coast, Queensland 4229 Australia

**Keywords:** Otitis media, Knowledge, Family practice, Primary health care, Qualitative research, Antibiotics

## Abstract

**Background:**

Acute otitis media is a common reason for antibiotic prescribing, despite strong evidence that antibiotics provide minimal benefit. Studies have demonstrated that patients’ (or parents’) expectations of antibiotics often influence general practitioners’ (GPs) decision to prescribe antibiotics, but few have explored parents’ expectations of the management of infections in children, or which factors influence the development of these expectations. This study aimed to explore parents’ knowledge and beliefs about the management of acute otitis media in children.

**Methods:**

Individual semi-structured interviews were conducted with 15 parents of children who had recently presented to their GP with acute otitis media. Parents were recruited at childcare centres or playgroups in Brisbane, Australia.

**Results:**

Many parents did not have an accurate understanding of what causes acute otitis media. GPs were primarily consulted for the management of symptoms such as pain and fever. Others specifically wanted reassurance or were concerned about hearing loss. Most parents assumed that antibiotics were the best treatment option. Parents’ perceptions about the best treatment were mainly based on their previous experience and the advice of the GP. Pain relief medications, such as paracetamol and non-steroidal anti-inflammatory drugs, were not considered by parents to be sufficient treatment on their own.

**Conclusion:**

There is discrepancy between parents’ beliefs and expectations of management of acute otitis media and the evidence-based recommendations. This study provides insights into parents’ expectations of management of acute otitis media, which may help inform clinicians about perceptions and misperceptions that may be valuable to elicit and discuss.

## Background

Acute otitis media is a common community-acquired infection and the majority of children will experience at least one episode of it during childhood. Children recover spontaneously without treatment, and antibiotics only have a modest overall benefit on the clinical course of acute otitis media. Twenty children have to be treated with antibiotics to prevent one child suffering ear pain after 2–7 days – that is, a number needed to treat to benefit of 20 [1]. The benefits of antibiotics are in the same order as the mild adverse effects, such as diarrhoea and candidiasis [2], which have numbers needed to harm of 14 [1]. Antibiotics seem to be most beneficial in children younger than 2 years of age with bilateral acute otitis media, and in children with both acute otitis media and otorrhoea [3].

Decisions about whether to use antibiotics need to weigh up their benefits with their harms, as prescribing leads to antibiotic resistance [4]. Antibiotic resistance is an increasing public health concern, not only at a population level, but also for individual patients, for whom use of antibiotics can result in bacterial resistance to that antibiotic for up to 12 months [5]. Although, current guidelines recommend withholding antibiotics in most cases of children with acute otitis media, many are still treated with antibiotics [6]. Antibiotic prescribing rates vary considerably between countries from less than 50 % in Dutch primary care [7] to more than 80 % in primary care in the UK [8]. One of the likely drivers of antibiotic overuse is demand, perceived or real, from parents. It is well-known that patients (or parents’) expectations of antibiotics influence general practitioners’ (GPs) decision to prescribe them [9]. Patients who are perceived by clinicians as expecting an antibiotic are 10 times more likely to be prescribed one [10]. However, there has been little exploration into parents’ expectations of the management of acute otitis media in children and what influences the development of these expectations. GPs need to be aware of parents’ knowledge and expectations to facilitate optimal communication during the consultation. The aim of this study was to explore parents’ knowledge and beliefs about the management of acute otitis media in children.

## Methods

### Participants

In this qualitative study, a convenience sample of parents was recruited from 3 childcare centres and 2 playgroups in Brisbane, Australia. Parents of children younger than 5 years, who had presented to a GP with acute otitis media in the last 6 months, were invited to participate. We gathered socio-demographic information (age, gender, education level and occupation), as well as a measure of their health literacy (the Rapid Estimate of Adult Literacy in Medicine (REALM)) [11]. All parents gave written informed consent and ethical approval was granted by Bond University Human Research Ethics Committee.

### Data collection and analysis

One of the researchers (JH) completed face-to-face semi-structured interviews with each parent. The interview guide was based on clinical experience, expert knowledge and relevant literature on patient expectations (Table 1). The interviews were audio-recorded and transcribed verbatim.

**Table 1:** Topics in the interview guide

Topics in the interview guide	
What do you think causes the illness?	
What treatments are available?	
Which treatment do you think is best?	
How do you know this?	
How does your GP know?	
Do you think ‘no antibiotic treatment’ is an option?	
In what ways can you decide which treatment is the best?	

The 5-stage framework approach [12] was used to analyse the textual data; a) reading and re-reading transcripts until familiar with the experiences, perspectives and language of each participant; b) creating a thematic framework which reflected the data and the interview questions; c) coding the data line-by-line against the themes and emerging sub-themes of the framework; d) summarising data into the framework, and; e) refining, comparing and interpreting themes. Two researchers (MPH, TH) independently identified the themes and in a face-to-face meeting, themes were discussed until consensus was reached. Recruitment of participants continued until data saturation, i.e. no new themes emerged from the analysis. All analysis were performed manually without any software tools.

## Results

We interviewed 15 mothers with a mean age of 37 years (*SD* = 5, range 29 to 49). They had an average of two children; most (n = 12) had tertiary education and 4 had a health professional qualification (nursing or allied health). Participants had a mean REALM score of 65.53 (*SD* = .83, range 63 to 66), which indicates that all parents had a reading level above Grade 9. Table 2 shows characteristics of parents. Thirteen out of the 15 children were treated with per oral antibiotics for the most recent episode of acute otitis media (Table 3). A delayed prescription was offered to one family however antibiotics were initiated at the same day. The mean duration of interviews was 21 min (range 15 to 31 min). The results which emerged could be framed around 4 themes: causes of acute otitis media, reasons for consulting a GP, beliefs about treatments, and sources of knowledge.

**Table 2:** Characteristics of parents

Participant	Age (years)	Number of children	Tertiary education	Health professional qualification	REALM Score
1	40	2	No	No	63
2	41	2	Yes	No	66
3	49	6	No	No	65
4	39	2	Yes	No	66
5	39	2	Yes	No	66
6	37	3	Yes	No	65
7	31	1	Yes	No	66
8	41	4	Yes	No	65
9	33	1	Yes	No	66
10	35	3	Yes	Yes	66
11	32	1	Yes	Yes	66
12	35	1	Yes	Yes	66
13	36	2	No	No	66
14	29	2	Yes	No	65
15	35	2	Yes	Yes	66

REALM = the Rapid Estimate of Adult Literacy in Medicine

**Table 3:** Antibiotic treatment of children

Participant (parent ID)	Antibiotic treatment^a^
1	+
2	+
3	+
4	+
5	+
6	+
7	Delayed prescription^b^
8	+
9	+
10	+
11	No
12	No
13	+
14	+
15	+

^a^Per oral antibiotic treatment for the most recent episode of AOM

^b^Antibiotics were initiated at the same day

### Causes of acute otitis media

Various causes of acute otitis media were suggested by parents, with many not having an accurate understanding.

*“Absolutely no idea, I don’t even know what an ear infection is really, it’s just something that I’m told that my children have”* (Participant 1)

Some parents related the cause of acute otitis media to having a cold that develops into an ear infection.

*“I thought it was like an extended version of a cold. A cold gone wrong basically, or gone worse”* (Participant 6)

*“I’d say he probably had a cold beforehand which then turned into something more than that”* (Participant 9)

Acute otitis media was also associated with teething, ear wax congestion, swimming, dirty bathing water and the size or shape of the Eustachian tubes in children.

*“Teething maybe, build up of ear wax I think”* (Participant 1)

*“I think in our children they have been water related. They’ve been swimming, something hasn’t drained, a little germ has festered in the ear and it’s got out of all control”* (Participant 4)

*“I think it’s because the Eustachian tubes are straight at that age, so anything like mucus and saliva and things that they have a cold can quite easily make their way to their ears”* (Participant 12)

### Reasons for consulting a GP

Pulling at the ear, pain, fever, no appetite and sleep disturbance were the signs and symptoms most parents reported when they suspected acute otitis media. Parents predominantly attended the GP because they did not want their child to be in any pain or because they wanted reassurance. The concerns that parents desired reassurance for were related to the possibility of a serious infection (e.g. septicaemia) or that their child would require hospitalisation.

*“…you don’t like little ones to be in pain, so it is quite traumatising when they carry on. You know when you’re not really treating anything and just letting go. So it is quite emotional as well. I want it to be fixed right then and there”* (Participant 11)

*“Well it could go further down and they could get like a… I have heard of people getting, well even septicaemia from going into the blood systems”* (Participant 15)

*“Possibly end up in hospital on a rehydration drip, which she has done previously, when she was very little”* (Participant 4)

Some parents were also concerned that their child’s eardrum would perforate and many worried about long-term damage, especially hearing loss.

*“I don’t really want his eardrum to burst”* (Participant 5)

*“I think most mothers worry about their child’s hearing…”* (Participant 14)

*“I would probably say that it might have a long-term impact on hearing. Irreversible”* (Participant 13)

### Beliefs about treatments

Most parents believed that antibiotics were the best and only treatment for their child’s acute otitis media. Others thought that the treatment strategy should depend on the type of infection (that is, viral or bacterial origin) and a few parents were unsure about the treatment.

*“…each time the antibiotics have worked like magic.”* (Participant 4)

*“I kind of thought it was quite simple really, just had an ear infection and that I needed antibiotics”* (Participant 15)

*“I think first of all you need to find out whether it’s viral or bacterial and then follow the according treatment”* (Participant 11)

Parents’ perceptions about the best treatment were mainly based on their previous experience and the advice of the GP. However, responses from parents suggested that they were not fully informed and had not previously considered the options.

*“The only option we were given was antibiotics. Apart from that, I wouldn’t imagine that there’s much else”* (Participant 7)

*“Well the only thing I’ve ever been offered is antibiotics, so I don’t know of anything else”* (Participant 1)

*“No, it was just, ‘Here’s the antibiotics and off you go’”* (Participant 14)

A few parents would accept ‘no antibiotic treatment’ as an option, if their child’s infection was monitored and they had the back-up of being able to re-consult if needed.

*“Yes, I think in certain circumstances, but it would have to be monitored, so… that the infection is resolving and that they’re not going to have any hearing issues as a result”* (Participant 10)

*“I would try it [no antibiotic treatment], as long as my option would be then to go back in a few days to make sure that it hasn’t got worse…”* (Participant 3)

Other parents were more cautious about this option and feared long-term damage such as hearing loss if antibiotics were not used or wanted a fast recovery (believing antibiotics would provide this). The time pressure of recovery was related to the importance of getting back to work or being on holiday for some parents.

*“… I am thinking well if they don’t treat him, what if he goes deaf?”* (Participant 1)

*“Preferably not, not when it comes to ears. I don’t want to take that chance with my child’s hearing”* (Participant 2)

*“…he was in full-time day care and from my point of view I had to get it fixed. So we went and got it filled that morning”* (Participant 9)

*“We are on holidays, we are in a different environment, if antibiotic is a suggested option, I will certainly go down that track…”* (Participant 5)

Pain relief medication, such as paracetamol (acetaminophen) or non-steroidal anti-inflammatory drug (NSAID), were used by all parents, mainly for temperature reduction and pain relief. However, most parents did not think of symptomatic relief of paracetamol or NSAIDs as a sufficient treatment on their own and they believed that treatment (such as antibiotics) to cure the infection was also needed.

*“But if I knew straight away that it was an ear infection, then I wouldn’t bother with just Panadol or something like that, I would go straight to the antibiotics to help their body knock it”* (Participant 4)

### Sources of knowledge

All parents relied on and trusted the GPs’ knowledge and assumed it was based on recent research and continuing medical education such as reading medical journals or attending conferences. A few parents mentioned that they preferred GPs who had children themselves because they felt that they had a better understanding of managing sick children.

*“Well I actually hope he knows what’s best, because he’s done more studies that what I’ve done… yeah, I mean we put a lot of faith in our GP’s”* (Participant 3)

*“Well, one hopes that they’re [GP’s] trained better than us to have a lot more information than we do, and I suppose we have to trust that they have done the research and that they do know”* (Participant 13)

*…some GPs have children themselves and understand that children need to be seen straight away…* (Participant 6)

Most parents preferred to receive information from the GP when they had a sick child. However, three other major sources of information for this situation were identified; (a) the internet; (b) close family and social contacts, particularly participants’ mothers and (c) other health professionals such as ear, nose and throat specialists and pharmacists.

*“I probably get the most information from the GP and trust that that source of information is relevant to my child, because I think every child is different”* (Participant 5)

*“I probably go to ‘Google’ or ‘Doctor Google’, one of those”* (Participant 14)

*“…mum usually diagnoses anything for everyone before they even suspect there’s something wrong themselves”* (Participant 4)

*“…if I couldn’t get into a doctor or something, which happens quite regularly, I would maybe go to the pharmacist…”* (Participant 1)

While it was expected that GPs knew about the best treatment for acute otitis media, only four parents recalled receiving an adequate explanation about the evidence of the management options for acute otitis media.

*“…there [are] options presented as hypotheticals, but they [GP’s] will always come to a decision and say, look I think we need to do the medication*”… (Participant 5)

*“Well I know they [GPs] used to give antibiotics, but I’ve been explained by the GP that that’s not what they do anymore. That they [antibiotics] don’t seem to alter the course of the illness, they tend to just give them pain relief and wait and see what happens…”* (Participant 12)

Although most parents thought they could ask their usual GP for a thorough explanation, some stated that if they saw another GP or an after hours doctor they often felt as if the consultation was rushed and there was no time for explanations.

*“…because it is an emergency appointment it feels quite rushed, it’s in and out the door…”* (Participant 2)

## Discussion

### Summary

Many parents did not have an accurate understanding of what causes acute otitis media. Parents primarily consulted the GP for the management of symptoms such as pain and fever or reassurance about possible complications, with many believing that antibiotics were necessary to prevent complications and hasten recovery. Most parents preferred to receive information from the GP when they had a sick child, but also the internet, close social contacts and other health professionals were used as a source of knowledge. Few parents recalled receiving an adequate explanation about the options for management of acute otitis media or their benefits and harms. Most parents assumed that antibiotics were the best and only treatment option for acute otitis media. Parents’ perceptions about the best treatment were mainly based on their previous experience and the advice of the GP. Pain relief medications (e.g. paracetamol or NSAIDs) were not considered by parents to be sufficient treatment on their own.

### Limitations

All interviews were completed by one author, who was not a GP, to ensure both consistency and bracketing of bias. However, our sample was a convenience sample, relatively small, exclusively female, and all had a high reading ability and most had a tertiary education. This limits the transferability of these results. Expectations for antibiotics may have been even higher in a sample that included male parents and parents with lower reading ability, as accurate knowledge of antibiotic effectiveness is associated with female sex and higher education level [13]. As most participants in the study received antibiotics, exploration of these issues in parents who have managed acute otitis media in their children without antibiotics would be worthwhile.

### Comparison with existing literature

We found, that the GP was primarily consulted for the management of symptoms such as pain and fever or for reassurance. Other studies have also shown, that patients with upper respiratory tract infections seek symptom relief [14] and that patient satisfaction is associated with reassurance and gaining information about the course of the disease [15]. A recent systematic review by Lucas et al. identified that parents would accept clinicians’ decisions if they felt they had received a good evaluation, even where this differed from their expectations [16].

A Dutch study of an internet-based survey of parents, found that almost 90 % of parents knew that not every child with a fever needs antibiotic treatment [17]. In contrast, we found that most parents believed that antibiotics were the optimal treatment. However, our study specifically focused on children with acute otitis media, and not childhood fever in general. We found that parents’ concern about hearing loss was prominent and it is possible that this concern may heighten perceptions of the need for and hence expectations of antibiotics. This finding emphasises the importance of exploring parents’ beliefs and expectations when consulting with a child with symptoms of acute otitis media and managing the expectations and reassuring where appropriate. Parents’ perceived vulnerability of children to health threats, increase their desire to remove the risk of such threats, and seeking consultations for reassurance were also identified in the systematic review by Lucas and colleagues [16] – all of which influence clinician antibiotic prescribing.

In a recent randomised double-blind placebo-controlled trial, the duration of middle ear effusion was reduced in children with acute otitis media who received antibiotics [18], although concerns about the lack of patient-centred outcomes in this trial have been raised [19, 20]. More information is needed, both of the possible long-term benefits and harms of antibiotic treatment of children with acute otitis media, e.g. risk of hearing impairment and recurrence rates [21].

Expectations of antibiotics were quite high among the group of parents, and this was attributed by parents as a consequence of the past experience of GP advice and treatment. In general, a positive feedback loop seems to have been established as patients’ expectations of antibiotics are associated with receiving an antibiotic [9, 10] and this perpetuates expectations of them in subsequent illness episodes [22]. Breaking this feedback loop is important and the use of antibiotics reserved for those who will benefit the most [1]. The tendency of people to overestimate the benefit of medical interventions, which subsequently drives their expectations for them, has been shown across various treatments and tests [23]. The role of use of pain relief medication should be explained to parents. We found that many parents confused the use of antibiotics with the use of medicine to manage symptoms. This finding is in line with a study in Greece where almost 70 % of parents confused antibiotics with other medicines used for symptomatic therapy for children with upper respiratory infections [24].

### Implications

This study provides insights into parents’ knowledge and expectations of management of acute otitis media, which may help inform clinicians about perceptions and misperceptions that may be valuable to elicit and discuss. It is very important that GPs are aware of parents’ knowledge and expectations to ensure optimal communication during the consultation [25]. GPs often elicit expectations for antibiotics in an indirect manner [26], hence expectations are not clearly addressed and managed. Shared decision making has an important role to play here. As part of the shared decision making process, evidence is brought into the discussion and patients’ concerns and expectations are explicitly sought and discussed [27]. Since the benefits of antibiotics for acute otitis media may only marginally, if at all, outweigh the harms, communicating this to patients/parents has been shown to reduce their desire for antibiotics [28]. Future efforts should focus on optimising the GPs’ awareness of the need to elicit and manage expectations and develop skills in integrating shared decision making into the consultation.

## Conclusion

There is a discrepancy between parents’ beliefs and expectations of management of acute otitis media and the evidence-based recommendations. Most parents primarily consulted the GP for the management of symptoms such as pain and fever. Others specifically wanted reassurance or were concerned about hearing loss. Most parents assumed that antibiotics were the best treatment option and pain relief medications were not considered by parents to be sufficient treatment on their own.

Parents need more information about acute otitis media, particularly the management options, including the option of ‘no antibiotics’, and the empirical benefits and harms of each option.

## Abbreviations

GP: General practitioner
AOM: Acute otitis media
REALM: The Rapid Estimate of Adult Literacy in Medicine
NSAID: Non-steroidal anti-inflammatory drugs
